# The Bamboo-Eating Giant Panda (*Ailuropoda melanoleuca*) Has a Sweet Tooth: Behavioral and Molecular Responses to Compounds That Taste Sweet to Humans

**DOI:** 10.1371/journal.pone.0093043

**Published:** 2014-03-26

**Authors:** Peihua Jiang, Jesusa Josue-Almqvist, Xuelin Jin, Xia Li, Joseph G. Brand, Robert F. Margolskee, Danielle R. Reed, Gary K. Beauchamp

**Affiliations:** 1 Monell Chemical Senses Center, Philadelphia, Pennsylvania, United States of America; 2 Shaanxi Wild Animal Rescue and Research Center, Louguantai, China; Barnard College, Columbia University, United States of America

## Abstract

A growing body of behavioral and genetic information indicates that taste perception and food sources are highly coordinated across many animal species. For example, sweet taste perception is thought to serve to detect and motivate consumption of simple sugars in plants that provide calories. Supporting this is the observation that most plant-eating mammals examined exhibit functional sweet perception, whereas many obligate carnivores have independently lost function of their sweet taste receptors and exhibit no avidity for simple sugars that humans describe as tasting sweet. As part of a larger effort to compare taste structure/function among species, we examined both the behavioral and the molecular nature of sweet taste in a plant-eating animal that does not consume plants with abundant simple sugars, the giant panda (*Ailuropoda melanoleuca*). We evaluated two competing hypotheses: as plant-eating mammals, they should have a well-developed sweet taste system; however, as animals that do not normally consume plants with simple sugars, they may have lost sweet taste function, as has occurred in strict carnivores. In behavioral tests, giant pandas avidly consumed most natural sugars and some but not all artificial sweeteners. Cell-based assays revealed similar patterns of sweet receptor responses toward many of the sweeteners. Using mixed pairs of human and giant panda sweet taste receptor units (hT1R2+gpT1R3 and gpT1R2+hT1R3) we identified regions of the sweet receptor that may account for behavioral differences in giant pandas versus humans toward various sugars and artificial sweeteners. Thus, despite the fact that the giant panda's main food, bamboo, is very low in simple sugars, the species has a marked preference for several compounds that taste sweet to humans. We consider possible explanations for retained sweet perception in this species, including the potential extra-oral functions of sweet taste receptors that may be required for animals that consume plants.

## Introduction

We and others have argued that taste function and diet are intimately connected through coordinated evolutionary processes that fit one to the other [1], [2], [3], [4]. Specifically for sweet taste, animal species that routinely consume plants express taste preferences for simple sugars and have functional sweet taste receptors (T1R2+T1R3), whereas many species that do not consume plants (e.g., strict carnivores) often do not prefer sugars and during evolution have lost sweet taste receptor function through detrimental mutations [1], [2], [5], [6]. It is thought that selection to maintain sweet taste receptor function is due to the need to identify plants rich in calories that are provided by the presence of simple sugars. A parallel argument has recently been made for bitter taste in vertebrates: the number of functional bitter receptors and the potential for contact with plant-based toxic compounds (i.e., the amount of plant-based material in the diet) are positively correlated [3].

The giant panda (*Ailuropoda melanoleuca*) provides a test of this coordination hypothesis for sweet taste. On the one hand, giant pandas exclusively consume plants, and thus one might predict that they should have a functional sweet taste system. This hypothesis is consistent with genetic studies that predict that genes that encode the sweet taste receptor (T1R2 + T1R3) are intact and functional [7]. On the other hand, their diet, 99% of which consists of bamboo, a single plant species that has a low sugar content [8], might lead one to predict that they, like strict carnivores [1], [5], [6], [9], [10], have lost sweet taste function.

To test these contrasting hypotheses, we evaluated sweet taste preference and sweet receptor binding in detail in this species. Specifically, we investigated how the giant panda responds to various compounds known to be sweet to humans, using both behavioral taste testing and the heterologously expressed giant panda T1R2+T1R3 sweet taste receptor, and correlated their sweet taste behavior to receptor structure and function. We used the same approach that we and others have used to generate mixed-species human-mouse sweet taste receptor pairs, which helped determine the monomer that is required for receptor sensitivity toward noncaloric human-specific sweeteners [11], [12], [13], [14], [15]. This study, part of a larger effort combining behavioral and molecular studies to define and relate taste receptor structure and function among species [1], [6], [16] shows that the giant panda has a marked preference for several compounds that taste sweet to humans, despite the fact that the species' main food, bamboo, is not at all sweet to humans.

## Materials and Methods

### Animals

Eight giant pandas between 3 and 22 years of age were studied at the Shaanxi Wild Animal Rescue and Research Center in China during a six-month period. This study was carried out in strict accordance with the recommendations in the Guide for the Care and Use of Laboratory Animals of the National Institutes of Health. The protocol was approved by the Monell Chemical Senses Center Institutional Animal Care and Use Committee (Permit Number: 1112). Giant Pandas were tested according to the protocol and with the permission and oversight of the director and staff of the Shaanxi Wild Animal Rescue and Research Center.

### Behavioral experiments

The animals were housed in groups but were tested individually and had access to food and water prior to behavioral taste testing. We used a two-bowl preference test that is adapted from the standard two-bottle preference test to assess giant pandas' preferences for tastants [16]. Two bowls, one containing 1 liter of plain water and the other containing the taste compound dissolved in 1 liter of water, were placed side by side by firmly attaching them to concrete holders, to avoid spillage. The animal could drink from either bowl for five minutes, beginning at 9:30 a.m. Every concentration of each tastant was tested twice (see below for a description of the taste stimuli); to avoid a side preference bias, the contents of the two bowls were reversed for the second tests. Each day of testing was followed by two rest days in which the animals received only tap water in both bowls. The five-minute test period and rest schedule were chosen to minimize potential postingestive effects. Intake was quantified by measuring the amount of fluid in each bowl. Wilcoxon matched-pairs signed rank tests were performed by comparing taste solution intake and water intake.

### Selection of sweeteners

Six natural sugars (fructose, galactose, glucose, lactose, maltose, and sucrose), five artificial sweeteners (acesulfame-K, aspartame, sodium cyclamate, neotame, and sucralose), and one sweet taste inhibitor (lactisole) were selected for the behavioral experiments. Lactisole (2.5 mM) was tested for its sweet taste suppression effect by adding it to 100 mM sucrose. Acesulfame-K, cyclamate, fructose, galactose, glucose, lactisole, lactose, maltose, sucrose, and sucralose were purchased from Sigma. Aspartame was purchase from Lab Safety Chemical. Neotame was a gift from the NutraSweet Company. The six sugars were chosen because they are commonly present in many fruits and plants and are major sweet-tasting ingredients found in many species' natural diets, and because they have been tested previously in other Carnivora species [16]. The five artificial sweeteners were selected because they also have been tested in other Carnivora species [16], as well as many other mammalian species [17], [18], [19], [20], [21], [22], and they are chemically and structurally diverse.

### Cloning of the giant panda T1R2 and T1R3 genes

To obtain giant panda (gp)T1R2 and gpT1R3 expression constructs, the full protein-coding sequences of gpT1R2 (NCBI access no. XM_002926831.1) and gpT1R3 (NCBI access no. XM_0029828.1) were chemically synthesized (Biomatik USA), and codon optimization was performed for expression in human-derived HEK293 peak rapid cells. To clone gpT1R2, a KpnI site and the Kozak sequence were introduced at the 5′ end before the start codon. To clone gpT1R3, an EcoRI site and the Kozak sequence were introduced at the 5′ end before the start codon. A NotI site was introduced at the 3′ end after the stop codon of both gpT1R2 and gpT1R3 to facilitate cloning into the expression vector pcDNA3.1. The integrity of all DNA constructs was confirmed by DNA sequencing. Human (h)T1R2 and hT1R3 were cloned as described previously [11].

### Stable cell lines

gpT1R2, gpT1R3, and Gα16-gust44, a chimeric G-protein to couple the receptor activation to calcium mobilization as readout [11], were cloned into pCDNA3.1 vectors with neomycin, zeocin, and hygromycin as selection drugs, respectively. After cotransfection into HEK293 cells, the resistant clones were selected and expanded clonally, and positive clones were established by examining responses toward sucralose, which elicits the most robust response in cells transiently transfected with gpT1R2+gpT1R3 and Gα16-gust44. Stable lines expressing gpT1R3 and Gα16-gust44 or parental cells were used as negative controls. The response profile of stable clones matched that of the transiently transfected cells with enhanced activities.

### Functional assay

HEK293-derived peak rapid cells were cultured at 37°C in Opti-MEM (Invitrogen), supplemented with 5% fetal bovine serum. In transient transfection cases, cells for calcium imaging were seeded onto 96-well plates at a density of 50,000 cells per well and were cotransfected using Lipofectamine 2000 (Invitrogen) with plasmid DNAs encoding human and/or giant panda T1Rs and Gα16-gust44 (0.06 μg/well for each plasmid) or controls (Gα16-gust44 + pcDNA3.1 or Gα16-gust44 and one of T1Rs). After 20 h, the medium was changed once; after an additional 24 h, the cells were washed with Hanks' buffered salt solution supplemented with 10 mM HEPES (HBSSH), loaded with 50 μl 3 μM Fluo-4AM (Molecular Probes) in HBSSH, incubated for 1 h, and then washed three times with HBSSH and maintained in 50 μl HBSSH. The plates were then placed into a FlexStation 3 system (Molecular Devices) or imaged under microscopy to monitor the fluorescence change (excitation, 494 nm; emission, 516 nm; cutoff, 515 nm) after the addition of 50 μl HBSSH supplemented with 2× tastants. Stable cells expressing gpT1R2 and gpT1R3 along with Gα16-gust44 were seeded onto 96-well plates at a density of 50,000 per well 24 h prior to assays. The assay procedure was the same as that of transiently transfected cells. The compounds tested behaviorally were examined in cell-based assays, but the concentrations differed in some cases because cell-based systems cannot tolerate the osmolarity of more concentrated taste solutions. For FlexStation traces, calcium mobilization in response to tastants was quantified as the percentage of change in fluorescence (peak fluorescence – baseline fluorescence level, denoted as ΔF) from its baseline fluorescence level (denoted as F) [11]. All data were collected from three independent experiments. Stable cells expressing gpT1R3 alone coupling Gα16-gust44 were similarly profiled for their responses to sugars and artificial sweeteners.

## Results

### Analyses of preference for sweet taste

**Figure 1:**
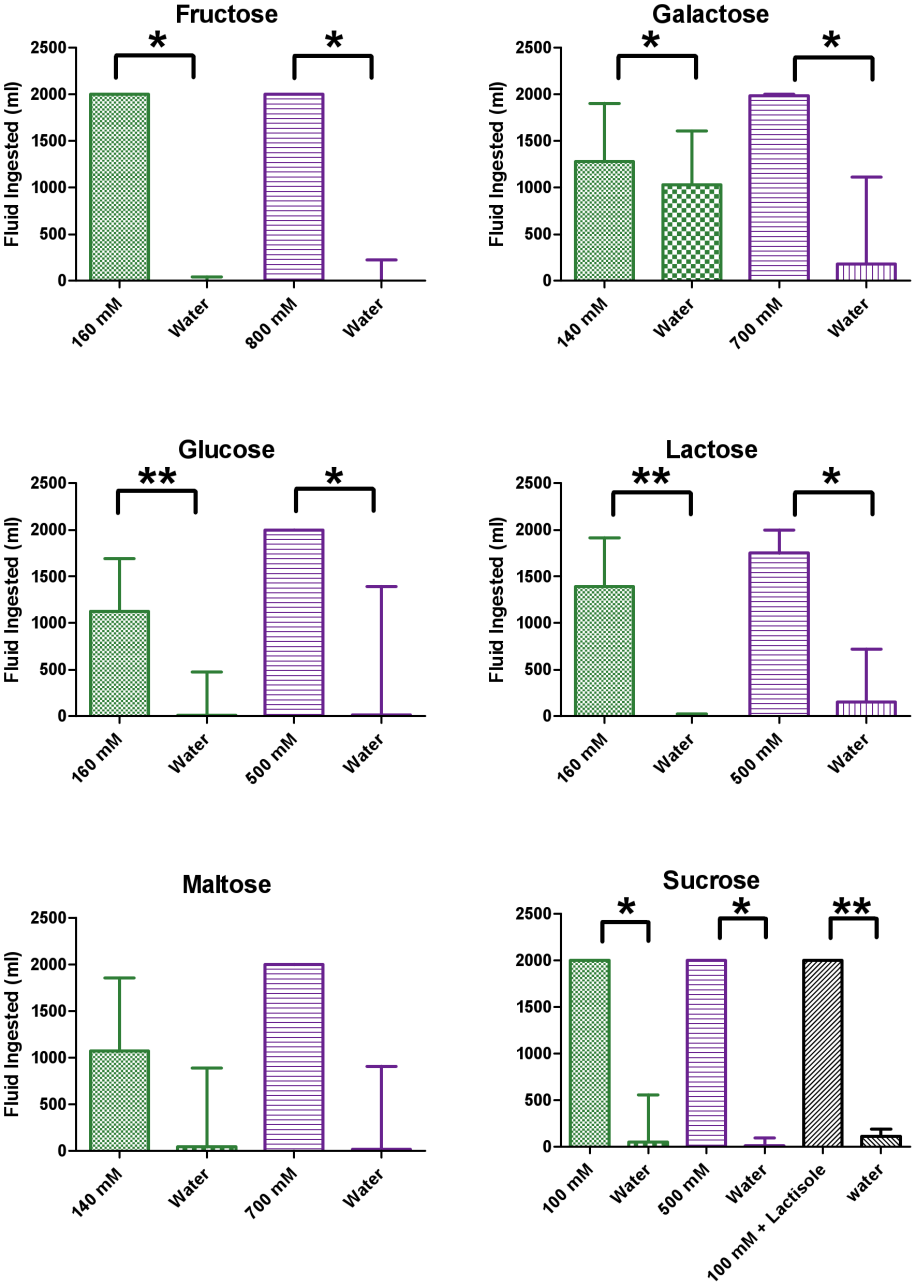
Giant panda preferences for sugars that taste sweet to humans. Eight giant pandas were tested behaviorally for their preferences for different concentrations of sugars using a two-bowl preference test: one bowl contained a tastant solution (1 L), and the other contained plain water (1 L). They were also tested for 100 mM sucrose plus the sweet taste inhibitor lactisole (2.5 mM; A). All tastant solutions were tested twice, on separate days. Each bar shows the median + interquartile range of the combined amount of a tastant or water consumed during the two separate tests for giant pandas: green for the lower concentration of tastant, and blue for the higher concentration (see Table S1 for detailed statistics). Only four pandas were tested for 700 mM maltose. Wilcoxon matched-pairs signed rank tests were performed for every tastant (vs. water) and for sucrose plus lactisole versus sucrose. *p<0.05; **p<0.01.

Giant pandas avidly consumed and strongly preferred most natural sugars, showing preferences for each the six natural sugars tested (at both concentrations) over water (Fig. 1, Table S1). Notably, giant pandas appeared to show high sensitivity to fructose, avidly preferring even a moderate concentration of 160 mM (p = 0.012); in all testing sessions, they finished the entire 1 liter of fructose solution and drank very little water. In contrast, giant pandas only modestly preferred galactose, even at the higher concentration of 700 mM (p = 0.0391). Giant pandas appeared to prefer maltose at both concentrations, but intake did not differ significantly from that of water; small sample size for the high concentration of maltose makes statistical testing impossible. The preference of the giant pandas for sucrose was not affected by the sweet blocker lactisole (p = 0.500).

**Figure 2:**
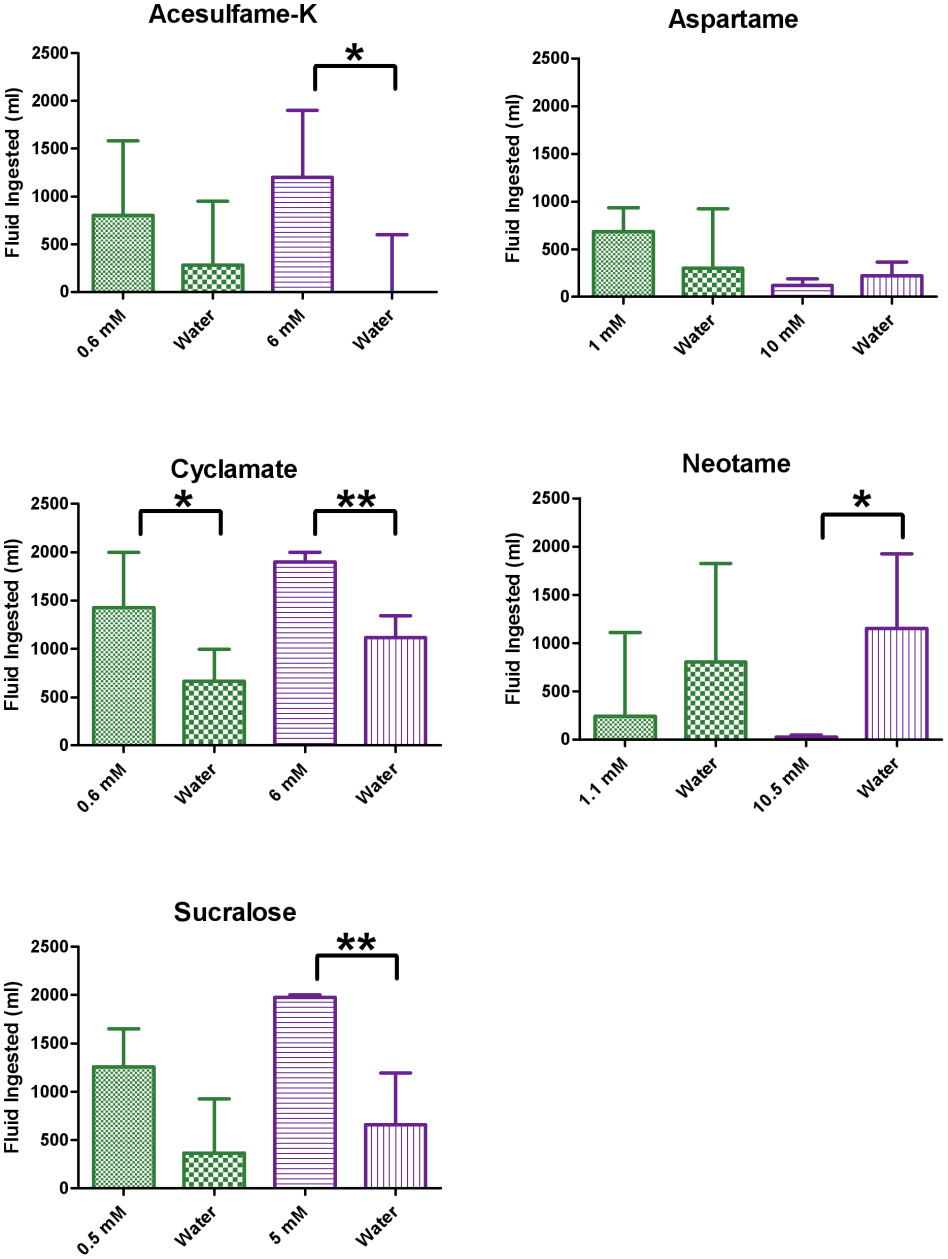
Giant panda preferences for artificial sweeteners that taste sweet to humans. Eight giant pandas were tested behaviorally for their preferences for different concentrations of artificial sweeteners using a two-bowl preference test: one bowl contained a tastant solution (1 L), and the other contained plain water (1 L). All tastant solutions were tested twice, on separate days. Each bar shows the median + interquartile range of the combined amount of a tastant or water consumed during the two separate tests for individual giant pandas: green for the lower concentration of tastant, and blue for the higher concentration (see Table S1 for detailed statistics). Only seven pandas were tested for acesulfame-K. Wilcoxon matched-pairs signed rank tests were performed for every tastant (vs. water). *p<0.05; **p<0.01.

Giant pandas exhibited varied responses to the five artificial sweeteners tested (Fig. 2, Table S1). They showed no preference or aversion for aspartame at both concentrations and were indifferent to neotame at the lower concentration but avoided it at the higher concentration (p = 0.0207). Giant pandas had a weak preference for cyclamate at the lower concentration (p = 0.0391) and had modest preferences for sucralose (p = 0.0078), acesulfame-K (p = 0.0156), and cyclamate (p = 0.0078) at the higher concentrations.

### Analyses of the giant panda T1R2+T1R3 sweet taste receptor *in vitro*

Expression constructs encoding the gpT1R2 and gpT1R3 receptor units were generated by transiently expressing gpT1R2 and gpT1R3 in HEK293 cells with a coupling chimeric G-protein (Gα16-gust44), which linked the receptor activation to calcium mobilization. In this system, the gpT1R2+gpT1R3 sweet receptor consistently responded to sucrose and sucralose but showed little response to other sweet-tasting compounds tested (Fig. S1A). As a negative control, sucrose and sucralose did not activate the cells transfected with Gα16-gust44, gpT1R2, or gpT1R3 alone (Fig. S1B. As a positive control, all sweeteners activated cells expressing hT1R2 or hT1R3 (Fig. S1C). Our *in vitro* data indicate that the giant panda sweet taste receptor is unequivocally receptive to sucrose and the artificial sweetener sucralose.

**Figure 3:**
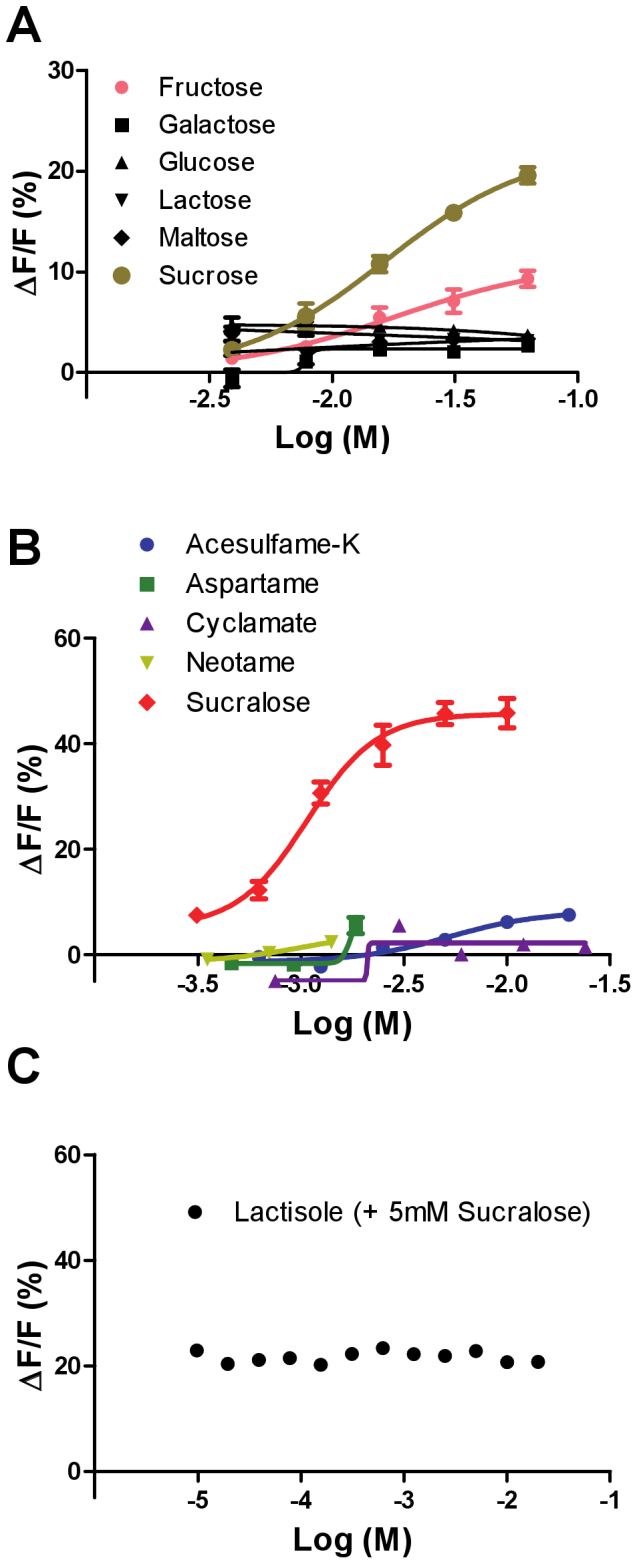
Responses of the giant panda sweet taste receptor T1R2+T1R3 to sugars and artificial sweeteners. T1R2+T1R3 was stably expressed in HEK293 cells along with Gα16-gust44. The receptor-expressing cells were then assayed by calcium mobilization for their dose-dependent responses to sugars (A), artificial sweeteners (B), and the sweet inhibitor lactisole (C). Data are expressed as percent change in fluorescence (ΔF  =  peak fluorescence – baseline fluorescence) from baseline fluorescence (F). The values represent the mean ± SEM of ΔF/F for five or six independent responses.

Because our assays using transient transfection showed small responses, we generated stable cell lines expressing gpT1R2 and gpT1R3, along with the coupling chimeric G-protein Gα16-gust44. The stably expressed receptor showed dose-dependent responses to sucrose and fructose but no detectable responses to galactose, glucose, lactose, or maltose, up to the highest concentrations (75 mM) that we can test in this assay without invoking nonspecific responses (e.g., due to osmolarity) (Fig. 3A). Furthermore, the stably expressed receptor showed dose-dependent responses to artificial sweeteners sucralose and acesulfame-K with varying efficacy but no detectable responses to cyclamate up to 24 mM (Fig. 3B) and no responses to aspartame and neotame up to 1 mM (Fig. 3B). At higher concentrations, aspartame and neotame elicited small, nonspecific responses in the stably transfected cells that expressed gpT1R3 or cells transfected with Gα16-gust44 alone (Fig. S1B). No other sweeteners activated Gα16-gust44–transfected cells at the highest concentration used in this study. The sweet blocker lactisole did not change receptor activity toward 5 mM sucralose (Fig. 3C).

### Mixed human + giant panda sweet taste receptors

**Figure 4:**
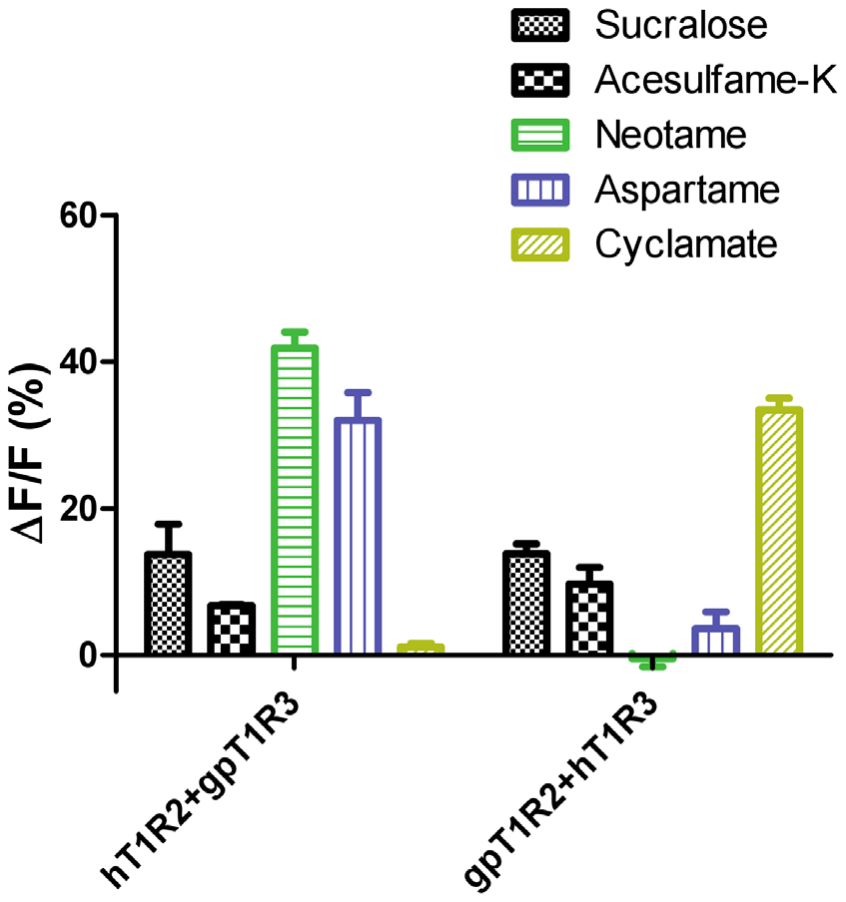
Responses of mixed-species receptors to artificial sweeteners. Human/giant panda mismatched receptor pairs (hT1R2+gpT1R3 or gpT1R2+hT1R3) were transiently expressed in HEK293 cells along with a reporter G-protein (Gα16-gust44), and their responses to artificial sweeteners were assayed by calcium mobilization: sucralose, 5 mM; acesulfame-K, 6 mM; neotame, 10 mM; aspartame, 10 mM; cyclamate, 6 mM. Data are expressed as percent change in fluorescence (ΔF  =  peak fluorescence – baseline fluorescence) from baseline fluorescence (F). Data were normalized to the responses of cells that expressed only Gα16-gust44. The values represent the mean ± SEM of ΔF/F for three independent responses.

We tested the hT1R2+gpT1R3 receptor pair and the gpT1R2+hT1R3 receptor pair for their responses to the panel of sweeteners (Fig. 4). The hT1R2+gpT1R3 receptor pair responded to sweeteners predicted to bind to the VFTM domain of hT1R2 (aspartame, neotame) [14], [15], [23], but not to cyclamate, which is predicted to bind to the transmembrane domain of hT1R3 [14], [24], [25]. Conversely, we found that the gpT1R2+hT1R3 receptor pair responded to cyclamate robustly but responded weakly or not at all to aspartame and neotame.

## Discussion

We approached this study with two competing hypotheses: (1) the plant-based diet of the giant panda would lead to the prediction that they, like all other plant-eating mammals tested, have fully functioning sweet taste perception, whereas (2) their almost total reliance on a plant diet lacking in simple (sweet) sugars would lead to the prediction that they have lost sweet taste function, as have obligate carnivores. This latter hypothesis was reinforced because the giant panda has lost the function of a similar receptor for amino acids and savory tastes that have been suggested to be associated with meat [7], [26].

Our data strongly support the first hypothesis. We found that giant pandas are strikingly similar to humans and many other mammals in their general preference for sugar plus water over plain water and are also similar in their liking for different types of sugars. Like humans, they strongly prefer sucrose and fructose and weakly prefer galactose, glucose, maltose, and lactose at low concentrations [27]. Our interpretation of these data is that fructose and sucrose are potent sweeteners at low concentrations and that higher concentrations elicit a strong preference. However, giant pandas differ from humans in their lack of a clear and consistent preference for many artificial sweeteners compared with plain water. A few sweeteners tested appear to be preferred by giant pandas (sucralose, acesulfame-K, and cyclamate), neotame was avoided at the higher concentration, and aspartame was neither preferred nor avoided. Due to unknown reasons, only a limited amount of water was ingested while paired with the high concentration of aspartame.

There are several potential explanations for the survival of the sweet receptor in giant pandas. Perhaps there is a currently unknown compound in bamboo that, although not perceived as sweet by humans (to humans bamboo does not have a perceptible sweet taste), does activate the giant panda sweet receptor. In light of the large differences in the compounds that activate sweet receptors across plant-eating species [17], [19], [20], [22], this hypothesis merits investigation. Second, perhaps giant pandas have sufficient opportunity to consume foods that taste sweet to humans, such as sugar cane, to maintain selection against loss of function; this explanation seems unlikely given what is known of the giant panda diet (comprising 99% bamboo) [28]. Third, perhaps loss of sweet receptor function has not happened due to stochastic processes; that is, by chance, no detrimental mutation has yet been fixed. This explanation is hard to test but may also account for why certain obligate carnivores such as ferrets retain sweet perception [16]. Finally, extra-oral functions of sweet taste receptors (e.g., in the gut or pancreas [29], [30]) may be required for animals that consume only plants even if these plants have no components that activate oral sweet receptors, and this maintains selection against loss of function. This is an attractive explanation because the presence a putative cellulose-metabolizing gut microbiome in the giant panda may help digest cellulose into simple sugars that do taste sweet [31].

In cell-based systems, we found that the giant panda's sweet taste receptor generally responded to the same sugars preferred by the animal in two-bowl preference tests, especially for sucrose, fructose, and sucralose. However, there was a lack of concordance between the behavior tests and cell-based measures for some sweeteners; this may be due in part to the technical limitations of the heterologous system. One limitation is that less potent sweeteners must be offered at high concentrations in two-bowl preference tests, but those same concentrations cannot be used in cell-based assays because of nonspecific cellular responses. There may be other explanations besides technical limitations that account for discrepancies between the *in vivo* and *in vitro* results, for instance, the existence of a second type of taste receptor, such as glucose transporters and ATP-gated K+ metabolic sensors [32].

Comparing cell-based and behavioral data can point to key regions of the receptor that bind a particular sweetener. For instance, neither the cells with gpT1R2+gpT1R3 nor the giant pandas themselves responded enthusiastically to aspartame. In fact, only humans and other Old World primates (but not New World primates or other mammals, with the odd exception of red pandas) apparently perceive it as sweet [16], [17], [18], [22]. Comparing the pattern of preference and the DNA sequence among species has localized the aspartame-sensitive region of the sweet receptor to the VFTM domain of hT1R2 [14], [15], [23]. Our current data also support this localization because when we co-expressed gpT1R2+hT1R3 in our *in vitro* assay, this pair of receptors showed no responsiveness. Likewise, lactisole acts on the sweet receptor in some species but not others, and its site of action has been localized to the T1R3 transmembrane domain [11], [13], [14]. Pandas were not affected by lactisole, which suggests that giant pandas must differ from humans at the key binding area of this monomer. In contrast, sucralose is a nearly universal sweetener for mammals, provided they have a functional T1R2+T1R3 sweet receptor [33]. Sucralose is predicted to interact with the T1R2 VFTM [34], so the region within this subunit must be highly conserved across species, including the giant panda. These are a few important examples of how species comparisons allow us to draw conclusions about the active sites of the sweet receptor for individual sweeteners.

There were several puzzling discrepancies in this study. Giant pandas had a weak preference for sodium cyclamate, but the gpT1R2+gpT1R3 receptor expressed *in vitro* was not activated by this sweetener. It may be that cyclamate is not potent enough to activate the cell-based system but does taste weakly sweet to the giant pandas. It is also possible that the weak preference for cyclamate is for the salty taste of the sodium or other sensory properties of sodium cyclamate. Supporting the latter hypothesis, as far as is currently known, only humans and closely related primates perceive sodium cyclamate as sweet [19], [20], [21], so it seems unlikely that the giant panda would be human-like in this regard. In addition, we know from other studies that sodium cyclamate interacts with the transmembrane domain of hT1R3 [14], [24], [25], but our present studies demonstrate that gpT1R3 does not interact and respond to sodium cyclamate, perhaps because of differences in key amino acids in the binding region [14], [24], [25]. Further work with cyclamate is warranted.

In conclusion, using a combination of behavioral and cell-based assay approaches, we demonstrate that the giant panda has a functional sweet taste system, and we characterized properties of this system. This study sheds light on the specificity and selectivity of the giant panda sweet taste receptor and provides data to correlate the structure and function of the sweet taste receptor with sweet taste behavior and possibly the dietary choices of the giant panda.

## Supporting Information

Figure S1 **Responses of the transiently expressed giant panda and human sweet taste receptor T1R2+T1R3 to sweeteners.** The giant panda gpT1R2+gpT1R3 (A) or human hT1R2+hT1R3 (C) receptors were transiently expressed in HEK293 cells along with a reporter G-protein (Gα16-gust44), and their responses to artificial sweeteners were assayed by calcium mobilization: acesulfame-K, 6 mM; aspartame, 10 mM; cyclamate, 6 mM; neotame, 10 mM; sucralose, 5 mM; sucrose, 62.5 mM. HBSS buffer was used as control. Data are expressed as percent change in fluorescence (ΔF  =  peak fluorescence – baseline fluorescence) from baseline fluorescence (F). The responses of cells that expressed only Gα16-gust44 (transiently transfected with Gα16-gust44 + pcDNA3.1) to sweeteners were shown in (B). 75 mM sucrose was tested in this case. The values represent the mean ± SEM of ΔF/F for three independent responses.
(TIF)

Table S1 Behavioral results for sweet taste preference.
(DOCX)
