# Supplementary figures and images for: The Bamboo-Eating Giant Panda (Ailuropoda melanoleuca) Has a Sweet Tooth: Behavioral and Molecular Responses to Compounds That Taste Sweet to Humans
Source: PLoS One. 2014 Mar 26;9(3):e93043. doi: 10.1371/journal.pone.0093043 (PMC3966865; doi:10.1371/journal.pone.0093043)

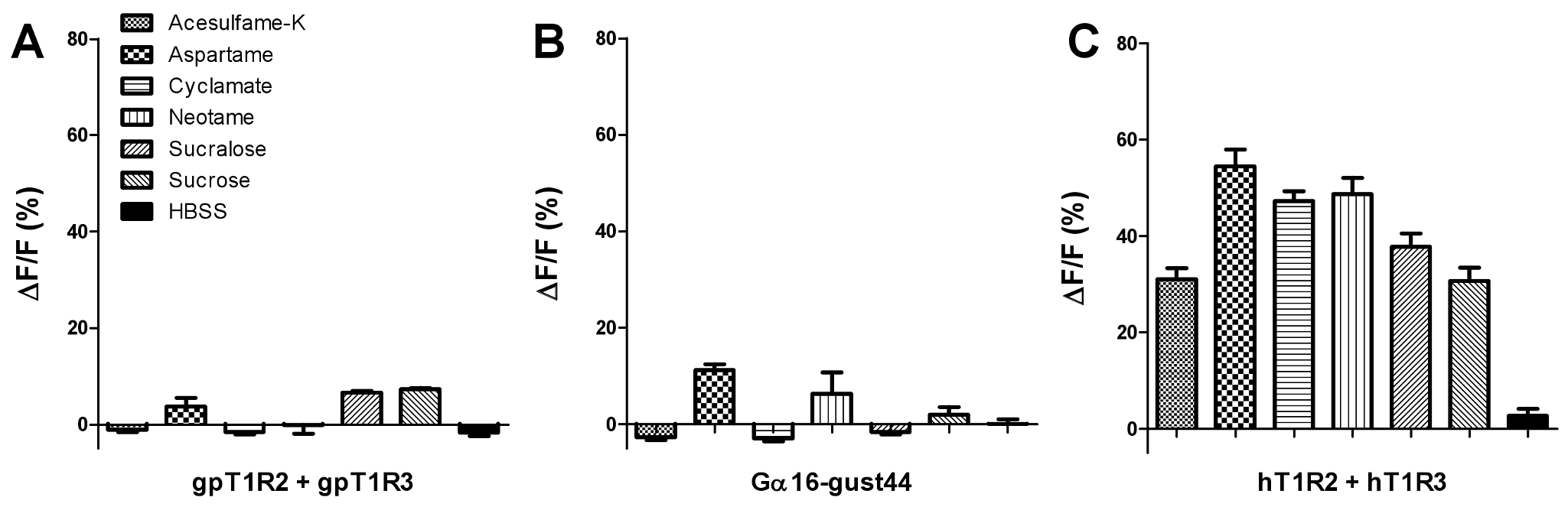

Supplement: Figure S1 — Responses of the transiently expressed giant panda and human sweet taste receptor T1R2+T1R3 to sweeteners. The giant panda gpT1R2+gpT1R3 (A) or human hT1R2+hT1R3 (C) receptors were transiently expressed in HEK293 cells along with a reporter G-protein (Gα16-gust44), and their responses to artificial sweeteners were assayed by calcium mobilization: acesulfame-K, 6 mM; aspartame, 10 mM; cyclamate, 6 mM; neotame, 10 mM; sucralose, 5 mM; sucrose, 62.5 mM. HBSS buffer was used as control. Data are expressed as percent change in fluorescence (ΔF = peak fluorescence – baseline fluorescence) from baseline fluorescence (F). The responses of cells that expressed only Gα16-gust44 (transiently transfected with Gα16-gust44 + pcDNA3.1) to sweeteners were shown in (B). 75 mM sucrose was tested in this case. The values represent the mean ± SEM of ΔF/F for three independent responses. (TIF) [file pone.0093043.s001.tif]
