# Supplementary material for: The Bamboo-Eating Giant Panda (Ailuropoda melanoleuca) Has a Sweet Tooth: Behavioral and Molecular Responses to Compounds That Taste Sweet to Humans
Source: PLoS One. 2014 Mar 26;9(3):e93043. doi: 10.1371/journal.pone.0093043 (PMC3966865; doi:10.1371/journal.pone.0093043)
Supplement: Table S1 — Behavioral results for sweet taste preference. (DOCX) [file pone.0093043.s002.docx]

**Table S1.** Behavioral results for sweet taste preference

| **Sweet compound** | ***n*** | **Amount consumed (ml)^a^** | | ***p*-Value** |
| --- | --- | --- | --- | --- |
|  |  | **Water + tastant** | **Water alone** |  |
| Sugars |  | (Minimum: 25% Percentile: Median:75% Percentile: Maximum) | (Minimum: 25% Percentile: Median:75% Percentile: Maximum) |  |
| Fructose, 160 mM | 8 | 2000: 2000: 2000: 2000: 2000^b^ | 0: 0: 0: 42.5: 370.0 | 0.01* |
| Fructose, 800 mM | 8 | 2000: 2000: 2000: 2000: 2000^b^ | 0: 0: 0: 225: 900 | 0.01* |
| Galactose, 140 mM | 8 | 310: 485: 1275: 1903: 1945 | 0: 20: 1030: 1605: 1790 | 0.03* |
| Galactose, 700 mM | 8 | 100: 1963: 1985: 2000: 2000 | 0: 105: 180: 1110: 1670 | 0.04* |
| Glucose, 160 mM | 8 | 520: 1028: 1125: 1690: 1900 | 0: 0: 10: 475: 950 | 0.008** |
| Glucose, 500 mM | 8 | 1500: 1993: 2000: 2000: 2000 | 0: 0: 15: 1390: 1480 | 0.01* |
| Lactose, 160 mM | 8 | 80: 955: 1390: 1915: 1950 | 0: 0: 0: 22.5: 1500 | 0.008** |
| Lactose, 500 mM | 8 | 80: 1195: 1750: 2000: 2000 | 0: 0: 150: 720: 1270 | 0.01* |
| Maltose, 140 mM | 8 | 100: 272.5: 1070: 1855: 2000 | 0: 0: 40: 887.5: 1600 | 0.15 |
| Maltose, 700 mM | 4 | 0: 500: 2000: 2000: 2000 | 0: 15: 907.5: 1200 | 0.20 |
| Sucrose, 100 mM | 8 | 0: 1250: 2000: 2000: 2000 | 0: 0: 50: 557.5: 800 | 0.02* |
| Sucrose, 500 mM | 8 | 2000: 2000: 2000: 2000: 2000^b^ | 0: 0: 10: 92.5: 160 | 0.01* |
| Sweeteners |  |  |  |  |
| Acesulfame-K, 0.6 mM | 7 | 0: 200: 800: 1580: 1850 | 0: 100: 280: 950: 1200 | 0.38 |
| Acesulfame-K, 6 mM | 7 | 250: 1000: 1197: 1899: 2000 | 0: 0: 0: 600: 900 | 0.02* |
| Aspartame, 1 mM | 8 | 140: 337.5: 685: 937.5: 1560 | 0: 62.5: 300: 925: 1460 | 0.56 |
| Aspartame, 10 mM | 8 | 10: 52.5: 120: 190: 1390 | 0: 52.5: 225: 365: 450 | 0.44 |
| Cyclamate, 0.6 mM | 8 | 1110: 1155: 1425: 1998: 2000 | 60: 270: 665: 997.5: 1700 | 0.04* |
| Cyclamate, 6 mM | 8 | 680: 1258: 1895: 2000: 2000 | 0: 460: 1115: 1343: 1600 | 0.008** |
| Neotame, 1.1 mM | 8 | 20: 85: 240: 1105: 1280 | 0: 90: 800: 1825: 2000 | 0.30 |
| Neotame, 10.5 mM | 8 | 10: 20: 25: 47.5: 120 | 0: 547.5: 1150: 1925: 2000 | 0.02* |
| Sucralose, 0.5 mM | 8 | 70: 255: 1255: 1650: 1980 | 100: 100: 365: 925: 1150 | 0.15 |
| Sucralose, 5 mM | 8 | 980: 1265: 1975: 2000: 2000 | 50: 107.5: 655: 1190: 1250 | 0.008** |
| Sweet taste inhibitor | 8 |  |  |  |
| Sucrose, 100 mM, + lactisole, 2.5 mM | 8 | 2000: 2000: 2000: 2000: 2000^b^ | Sucrose 100mM, 0: 1250: 2000: 2000: 2000 | 0.50 |

^a^Data show consumption across two tests per concentration, averaged across the number of pandas tested. p-Values were calculated by Wilcoxon matched-pairs signed rank tests for every tastant versus water and for sucrose plus lactisole versus sucrose. p-Values were rounded to the nearest hundredth with the exception of those less than 0.01. * p < 0.05, ** p < 0.01.

^b^All pandas consumed all of this tastant during each test.
